# scRNA sequencing technology for PitNET studies

**DOI:** 10.3389/fendo.2024.1414223

**Published:** 2024-07-24

**Authors:** Walaa Asaad, Marina Utkina, Anastasia Shcherbakova, Sergey Popov, Galina Melnichenko, Natalia Mokrysheva

**Affiliations:** Department of General, Molecular and Population Genetics, Endocrinology Research Centre, Moscow, Russia

**Keywords:** PitNETs, ScRNA-seq, pituitary tissue, pituitary development, PitNETs structure, PitNETs invasion, PitNETs immunity, tumor microenvironment

## Abstract

Pituitary neuroendocrine tumors (PitNETs) are common, most likely benign tumors with complex clinical characteristics related to hormone hypersecretion and/or growing sellar tumor mass. PitNET types are classified according to their expression of specific transcriptional factors (TFs) and hormone secretion levels. Some types show aggressive, invasive, and reoccurrence behavior. Current research is being conducted to understand the molecular mechanisms regulating these high-heterogeneous neoplasms originating from adenohypophysis, and single-cell RNA sequencing (scRNA-seq) technology is now playing an essential role in these studies due to its remarkable resolution at the single-cell level. This review describes recent studies on human PitNETs performed with scRNA-seq technology, highlighting the potential of this approach in revealing these tumor pathologies, behavior, and regulatory mechanisms.

## 1 Introduction

PitNETs, also anointed pituitary adenomas (PAs), are benign tumors originating from the anterior lobe of the pituitary gland. These tumors have a high incidence of between 3.9 and 7.4 cases per 100,000 per year (a mean annual incidence of approximately 5.1 cases per 100,000) (1–3). In general, PitNETs are classified based on tumor cell lineages identified by immunohistochemical analysis of the main three TFs (SF1, PIT1 and TPIT) and the blood serum levels of pituitary hormones secreted by pituitary tumor hormone-secreting cells (4). This clinicopathological classification categorizes the PitNETs into PIT1 lineage (lactotroph, somatotroph, thyrotroph, and silent PIT1 tumors), TPIT lineage (corticotroph and silent TPIT tumors), SF1 lineage (gonadotroph and silent SF1 tumors), null cell (expressing no TFs or hormones), and plurihormonal tumors. According to this classification, PitNETs are either functional (secreting) adenomas or Silent Pituitary Adenomas (SPAs), also called Non-functioning Pituitary Adenomas (NFPAs), which are non-secreting forms of pituitary adenomas, classified according to TF expression to silent PIT1, silent TPIT, silent SF1, or null cell tumors (5, 6). According to studies, NFPAs account for 30% of PitNETs. The majority of NFPAs (73%) belong to the SF1 lineage, 16% of them are silent corticotroph adenomas (SCAs) of the TPIT cell lineage, while PIT1 cell lineage silent adenomas account for 9% of NFPA cases, with a rare occurrence of truly null cell tumors (negatively stained tumors of TFs and pituitary hormones) (4, 7). Although these NFPAs do not cause a hormone hypersecretion syndrome, they do cause mass-related symptoms and signs, such as visual impairment, headaches, or hypopituitarism. Research is conducted to understand the pathology of NFPAs and differences in the gene expression profiles between NFPAs and secreting pituitary tumors are defined (8–10). A deeper understanding could lead to better treatment and, thus, improved patient outcomes. In general, the standard therapies of PitNETs include surgery, medication, and radiotherapy, and PitNETs are defined as aggressive or refractory if they are resistant to these therapies (4). One challenge in PitNET treatment is their recurrence (in 10-30% of cases) despite the optimal application of treatment protocols (11, 12). Although PitNETs are mostly benign, they may present invasive, aggressive, and malignant metastasis behavior, which raises the importance of more profound studying of their pathology. Integrating scRNA-seq technology into studies on PitNETs represents a paradigm shift in our understanding of these tumors, shedding light on their intricate molecular landscape and uncovering previously unrecognized cellular subpopulations and signaling pathways. This deeper understanding of these tissues will help us optimize the treatment protocols and prognosis prediction and outcomes of patients. Notably, other transcriptomic methods like genome-wide mRNA analysis (13), single nucleus RNA sequencing (snRNA-seq) (14) and spatial transcriptomic (15) were also applied for PitNETs studies. However, our review focuses on the scRNA-seq method of human pituitary studies. Through an overarching examination of the studies leveraging scRNA-seq in human PitNET research, this review provides a comprehensive overview of the current knowledge in this field.

## 2 scRNA sequencing technologies and applications

Since the publication of this technology in 2009 (16), it has been used to get a massive amount of information of different cell types and tissues (17). Applying scRNA-seq technology in tumor research affected different therapy protocols when it revealed new molecular processes in tumor progression or regulation. Considering the heterogeneous nature of PitNET tissues, the scRNA-seq technique has the potential for answering, on the molecular level, unsolved questions about the pathology, cell heterogeneity, behavior, recurrence, invasion, and immune features of PitNETs. Examples of these studies will be discussed further in our review, highlighting the importance of this accumulated knowledge for better future therapies and patient outcomes.

### 2.1 Workflow of scRNA sequencing method for pituitary gland tissues

**Figure 1:**
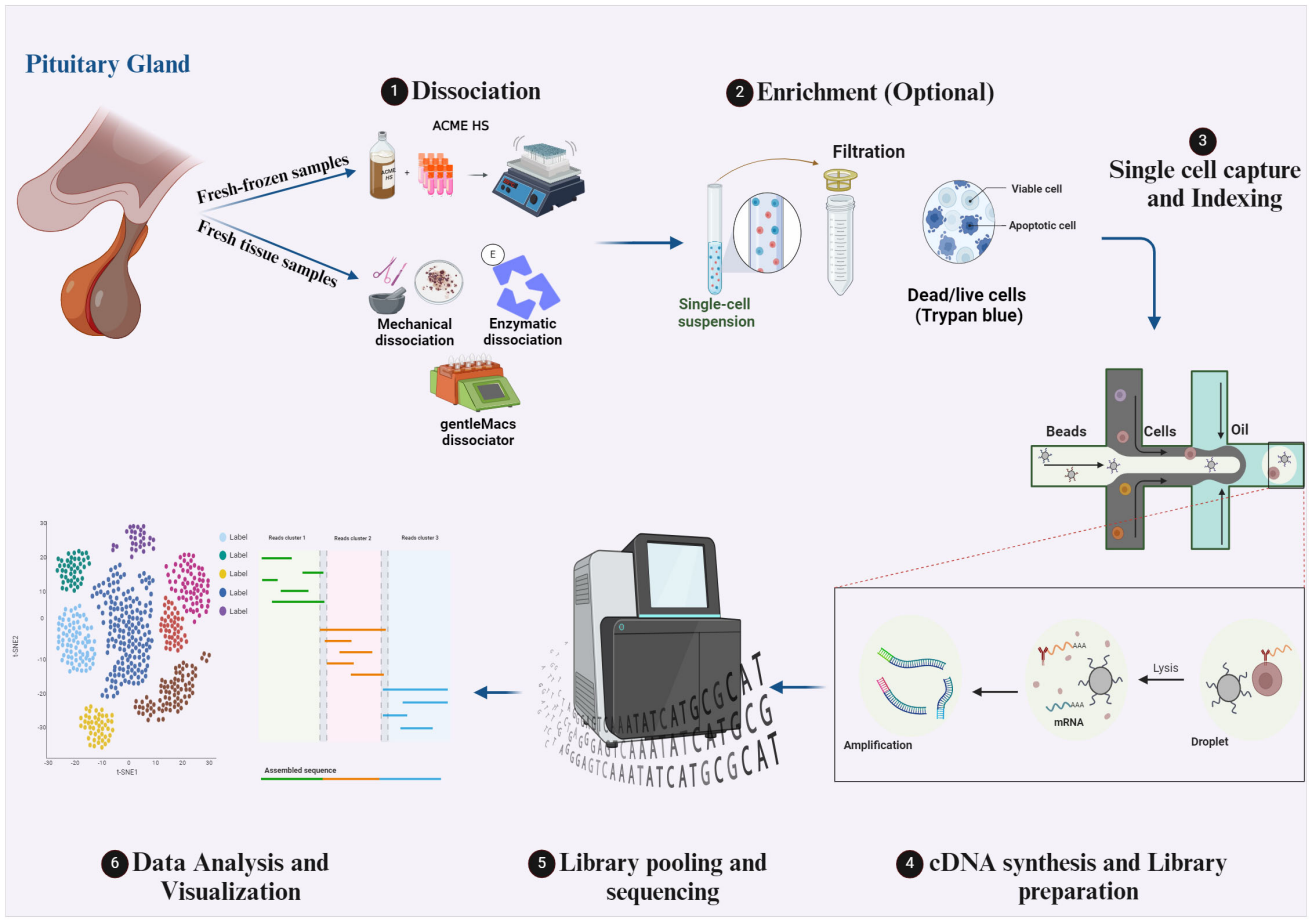
The workflow of scRNA-seq protocols for human pituitary gland tissues. Created with BioRender.com.

The technology of scRNA-seq is compelling since it allows the investigation of transcriptomes on the single-cell level. The maximum benefit of this method can only be obtained by using optimal protocols during the multi-step scRNA-seq procedure. Notably, different scRNA-seq protocols of different tissues have already been published (17, 18) and the choice of the protocol to apply depends on many factors, mainly the research question and the tissue under investigation. The workflow of the scRNA-seq protocol of pituitary tissues is illustrated in **Figure 1** of our review. Briefly, the first primary step of the scRNA-seq for pituitary specimens is to obtain a single-cell suspension. After surgical dissection of a pituitary specimen (fetal pituitary could be extracted under a dissecting microscope), dissociation of freshly obtained tissue is performed by mechanical “chopping” with a scalpel on a plate (19), by laser capture microdissection (LCM), by enzymatic digestion by collagenase II/IV (20–22), collagenase I/II/IV (23) or collagenase VIII (24) at 37 °C for 15-30 min, or by combinatorial enzymatic and mechanical methods like the gentleMACS dissociator and commercial Human Tumor Dissociation Kit from Miltenyi Biotec which is used in many studies (25–27). Notably, isolating intact cells for scRNA-seq from fresh-frozen solid tissues is challenging, which limits the application of the scRNA-seq method. However, in a recent study published by our laboratory we adapted the ACetic-MEthanol High Salt dissociation method – ACME HS to effectively isolate intact single cells from fresh-frozen endocrine tumor samples including pituitary tissue samples samples, which was not previously described, facilitating scRNA-seq studies for fresh-frozen specimens routinely collected from biobanks (28). The ACME HS method combines simultaneous acetic acid-based dissociation and methanol-based fixation to capture transcriptional profiles of whole individual cells from fresh-frozen tissue samples. Additionally, it uses a High-Salt (HS) washing buffer instead of standard PBS to prevent RNAse reactivation during rehydration. This technique aims to preserve cell morphology and RNA integrity, minimizing transcriptome changes and providing a better representation of mature mRNA. 

After the single-cell suspension preparation, an optional enrichment step could be conducted to choose a specific high-quality cell population and discard the unwanted (dead) cells. However, this step can be skipped when small tumor tissue is analyzed, and a significant cell count loss is expected, like a pituitary microadenoma tissue. The optional enrichment step could be performed by fluorescence-activated cell sorting (FACS) of a specific cell type in the tissue for further analysis. Filtration of the cell suspension by 40,45 or 70 µm filters is one of the most used methods for enrichment to remove cell aggregates, followed by pelleting the cells and resuspending them in PBS with 1% BSA (25) or in L15 with 10% FBS (20). Assessment of cell viability in the suspension by trypan blue exclusion (25), and the treatment of the single cell suspension with red blood cells lysis buffer (23) or a second round of enzyme digestion by different digestive enzymes like accutase (20), trypsin, trypLE or dispase to dissociate any remaining cell clusters are also used methods of enrichment. The next step is the single-cell capture and distribution to deliver each cell into an individual reaction tube (or well or chamber). Low throughput methods for cell capturing include mouth pipetting (22), after which cells are lysed and STRT-seq protocol for RNA transcripts capture by biotin labeling and Streptavidin Dynabeads might be applied, followed by cDNA synthesis and library preparation protocol which is described in (21, 22). High throughput methods for cell capture include FACS, magnetic-activated cell sorting (MACS), and microfluidic devices. In fact, droplet microfluidic methods are widely used for pituitary scRNA-seq studies (20, 23, 25–27). This method enables the generation of water-in-oil droplets and massively increases the throughput of single-cell sequencing. It also allows the combinatorial pre-indexing of RNA transcripts by co-encapsulating cells and barcode-containing beads or hydrogels so that all RNA from each cell can be labeled with a unique barcode. After pooling and sequencing, each read can be matched to the cell of origin. The main droplet-based single-cell sequencing methods are 10X genomics, InDrop, and Drop-seq, which differ in bead type, barcode design, and cDNA amplification (29). Next step is the next-generation sequencing of prepared libraries. Different sequencing platforms are available; the most common are Illumina sequencers by Sequencing By Synthesis (SBS) method, which accounts for > 90% of overall sequencing data and was used in all scRNA-seq articles mentioned in our review. The final foremost step is the scRNA-seq data analysis (30, 31). This step starts with raw data processing in which raw reads are cleaned, demultiplexed, mapped to the reference genome, and quantified to generate gene/barcode matrices. Then, a filtration step via QC covariants of cells in the count matrix is applied to avoid misinterpretation of ambient gene expression, apoptotic cells, and multiplets. After that, the raw expression in count reads is normalized to denoise and remove batch effects, followed by specific variable selection for further visualization. The next step is the linear dimensionality reduction of data based on selected features and designated genes. The last step is cell clustering and data visualization based on previous analysis step scores.

## 3 ScRNA-seq for studying the pituitary gland tissues

By enabling the comprehensive profiling of individual cells within a pituitary gland tissue, scRNA-seq technology provides insights into its cellular composition, gene expression patterns, and molecular diversity. The unclear molecular pathology and variable clinical features of PitNETs challenge their classification, and pose significant clinical difficulties in diagnostic evaluation and prognostication of these tumors (32). The different expression profiles of different cell types and lineages of PitNETs were previously investigated (33–35). However, integrating the scRNA-seq technology in these studies helped to overcome limitations and revealed previously undescribed markers, activated pathways, and cell interactions. Examples of these studies are discussed in the following parts.

### 3.1 Studying the normal pituitary cell types, lineages, and development processes

**Figure 2:**
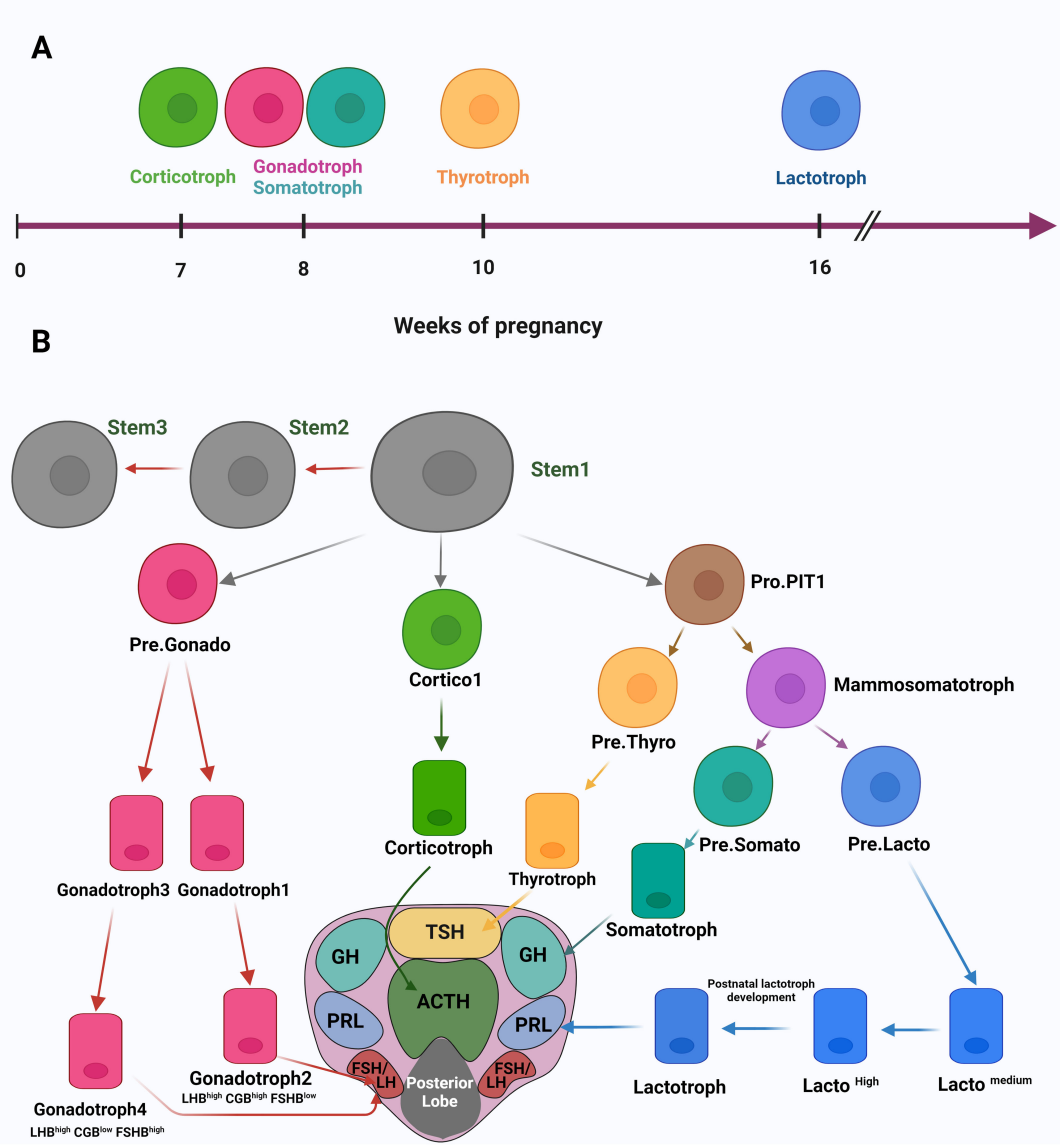
**(A)** A timing illustration of human embryonic development of pituitary cells. **(B)** A suggested developmental trajectory of the APG by integrating data from different articles. The developmental trajectory starts from the stem cell (Stem1) to the fully differentiated hormone-secreting cells (corticotrophs, lactotrophs, somatotrophs, thyrotrophs and gonadotrophs 2 and 4), indicated by arrows to their distribution in the horizontal section of the pituitary gland. Created with BioRender.com.

The first study published about using the scRNA-seq method for studying the human pituitary gland was in 2020 (22). In this study, the researchers analyzed fetal pituitary tissues to understand, on the molecular level, the development processes and the timing of endocrine cell differentiation. By analyzing scRNA data of 21 human fetuses from 7-25 weeks post-fertilization, they could identify 14 different cell clusters from anterior and posterior fetal pituitary tissue. Further analysis of these distinct cell clusters gave a timing understanding of pituitary cell differentiation, where corticotrophs are the first cell type to appear at seven weeks. Next, the gonadotrophs and somatotrophs appear at eight weeks, followed by thyrotrophs at ten weeks and lactotrophs at 16 weeks (**Figure 2A**). These results were proved by immunohistochemistry (IHC) and immunofluorescence assays in the same article. Notably, this article identified “Pro-PIT1_all cells” as a common progenitor for all three hormone-producing cell types of the PIT1 cell lineage (lactotrophs, somatotrophs and thyrotrophs), in addition to the thyrotroph precursor- Pre.Thy, and the potential precursor for somatotroph – Pre.Som. Moreover, the authors characterized stem cells clustered by Gene Set Enrichment Analysis (GSEA) and identified enriched pathways involved in cell proliferation, cell cycle, and Extracellular matrix (ECM) regulation. The power of scRNA-seq manifested when authors were able to identify three stem cell subpopulations STEM1, 2, and 3. These subpopulations differ in their enriched pathways for the induction and reduction of cell proliferation which indicate an embryonic state shift of the pituitary stem cells maintained by negative regulation of cell proliferation and adjustments in TFs and signaling pathways. Moreover, the authors characterized the cellular heterogeneity of the pituitary tissue and identified multistep developmental trajectories of gonadotrophs and corticotrophs. This includes the developmental trajectory Cortico1, then Cortico2 for the PIT-T lineage and Pre.Gonado then either Gonadotroph1 followed by Gonadotroph2 (LHB^high^CGB^high^FSHB^low^) or Gonadotroph3 followed by Gonadotroph4 (LHB^high^CGB^low^FSHB^high^) for the SF1 lineage (**Figure 2B**). Other precursor cells during pituitary gland development were identified later. In another study (20), the analysis of scRNA-seq expression data of hormone-secreting cells showed an expression similarity between lactotrophs and somatotrophs which support previously published data that somatotrophs and lactotrophs differentiate from the same precursor cells - the mammosomatotrophs. Corresponding with the scRNA article discussed above (22), the article by Zhang et al. (25), also identified the developmental trajectories of Pro.PIT1 cluster of the anterior pituitary gland (APG). In fact, merging the adult APG data with the fetal APG data, showed that large numbers of fetal PIT1 cells were in a poor differentiation status and similar to the Pro.PIT1 cells in the adult APG, while cells of APG of TPIT and SF1 lineages correspond with fetal cells. Integrating these findings from different articles helped us set a suggested developmental trajectory of the anterior lobe of the pituitary gland (**Figure 2B**). The developmental trajectory starts from the stem cell (Stem1) to the fully differentiated hormone-secreting cells (corticotrophs, lactotrophs, somatotrophs, thyrotrophs and gonadotrophs 2 and 4) crossing by intermediate precursor cells, all shown in **Figure 2B**.

### 3.2 Studying the PitNETs gene expression profiles and heterogeneity

Applying the scRNA-seq method to understand PitNETs pathology on the molecular level requires comparing tumor tissue cells and the corresponding matched pituitary normal cells. We can see this by examining the sample types in different studies. For example, two 2023 and 2024 studies included three adult pituitary gland tissues vs. 21 PitNETs and four normal pituitary tissues vs. 24 PitNET samples, respectively. This comparison identifies distinct gene expression profiles across normal and tumor cell types and lineages. For example, Cui et al. (21) found, by comparing Differentially Expressed Genes (DEGs) of tumor and normal cells, that most DEGs are downregulated in tumor gonadotrophs. In contrast, they are upregulated in tumor somatotrophs and lactotrophs. In fact, gonadotroph tumors are thought to be controlled by DNA methylation or other epigenetic mechanisms (36, 37), which, according to Cui et al., could explain the downregulation of DEGs of gonadotroph tumors (21). This article also defined *AMIGO2*, *ZFP36*, *BTG1*, and *DLG5* tumor-related genes. More tumor-related genes were identified by Zhang et al. (25), and their study expanded the molecular markers of each cell lineage since it identified *ENPP1*, *NTS*, *GATA3*, *IGFBP7*, and *LRRC4C* as specific markers for PIT1 lineage; *ID4* and *CITED1* as specific markers for TPIT lineage; and *FSHB* as a specific marker for SF1 lineage.

**Figure 3:**
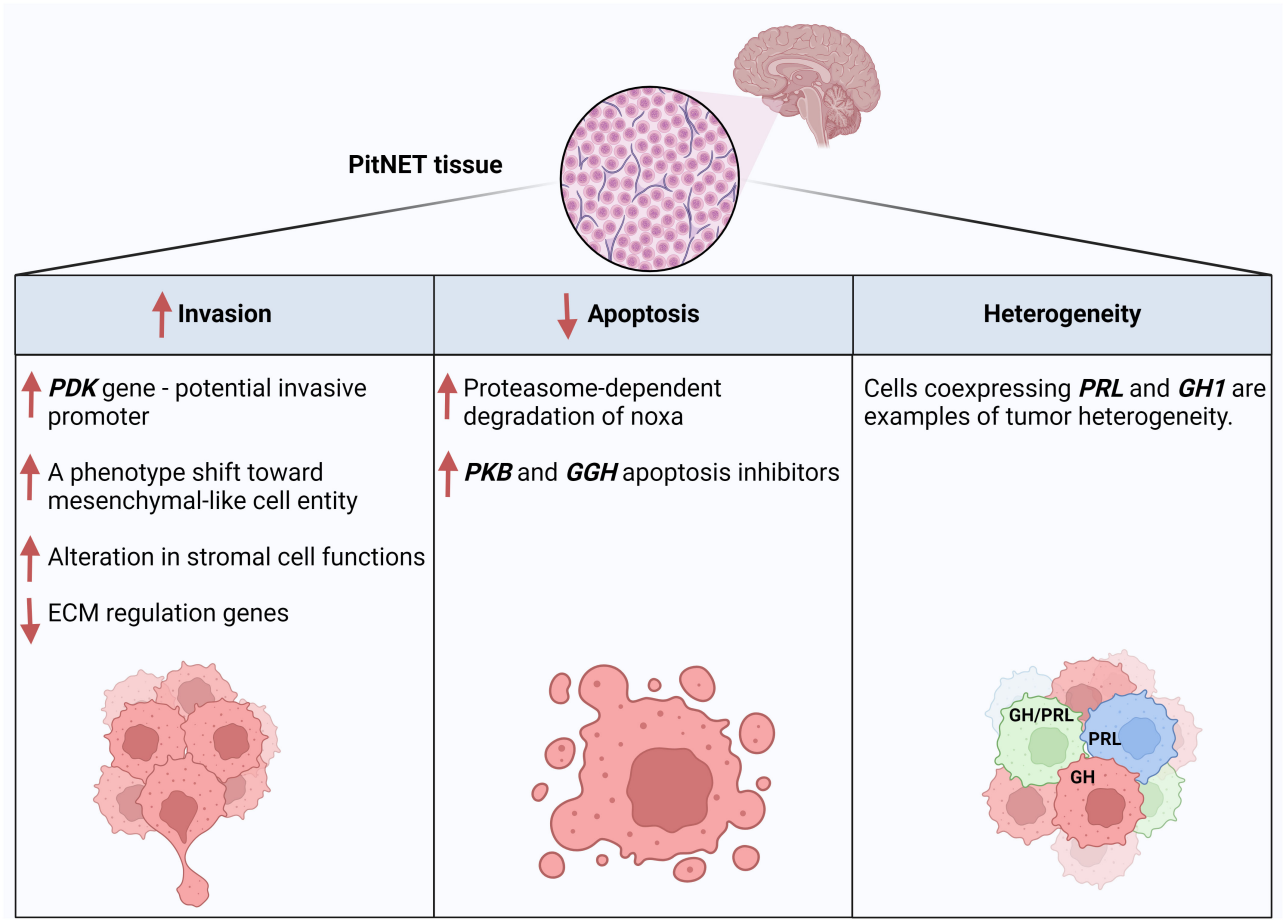
Examples of PitNET regulatory mechanisms and features revealed by scRNA-seq data analysis of PitNET samples. Created with BioRender.com.

scRNA-seq studies also helped us understand the heterogeneity of PitNETs. Applying single-cell multi-omics analysis, including scRNA-seq, revealed slight but apparent intra-tumor genomic heterogeneity in copy number variations (CNVs), according to Cui et al. (21). Notably, CNVs are expected in PitNETs (38, 39), and a recent scRNA-seq study (24) showed that the three PitNETs lineages show CNV events, with the PIT1 lineage having the highest one. In another study (20), scRNA data indicated intrinsic tumor heterogeneity since, compared to normal tissues, the gene expression profiles in tumor cells differed significantly from each other. The authors of this article compared tumor and normal cells and found that in normal tissues, many cells coexpress *GH1* and *PRL*, while most tumor cells only express one. However, cancer cells expressing both genes express them relatively higher than those in normal tissue. Thus, it was suggested that a PitNET heterogeneity could mainly be caused by the hormone-encoding genes and related regulatory genes **Figure 3**. Overall, this article highlighted intrinsic cellular heterogeneities of tumor cells and that the expression of hormone-encoding genes defined the significant variations of the PIT1-lineage tumor cell transcriptomic heterogeneities.

## 4 Studying tumor behavior and activated pathways (invasion, apoptosis and recurrence)

### 4.1 Invasion properties

About 35% percent of PitNETs are invasive (40), where the invasion of the cavernous sinus or erosion of the sellar floor and invasion of the sphenoidal sinus is detected during surgery, by radiological classification, or using MRI (41, 42). The invasive behavior of these tumors can limit the extent of resection and surgical outcome, especially in functional tumors (43). A study in 2020 (44) found an association between CD86+/CD163+ macrophage infiltration and gonadotroph tumor invasion behavior. Another study found that the cavernous sinus invasion (CSI) is affected by the transcription factor expressed in the tumor, with PIT-1-expressing tumors having a higher rate of invasiveness (43). Many researches are conducted to understand the pathology of invasive tumors and the critical differences between them and non-invasive tumors, and a recent study in 2023 (19) identified eight key genes (*BMP6*, *CIB2*, *FABP5*, *HOMER2*, *MAML3*, *NIN*, *PRKG2*, and *SIDT2*) that may play a crucial role in the invasive progression of NFPAs.

A study in 2022 (26) used scRNA-seq method for deeper investigation of SCAs by comparing them to their functioning counterparts, Functioning Corticotroph Adenomas (FCAs). scRNA-seq data analysis has shown that, compared with FCAs, a reduced expression is found in SCAs of essential proteases and their regulators involved in prohormone processing, like those included in POMC processing. Using Gene Ontology (GO) cellular component analysis by ENRICHR, it was shown that FCAs highly expressed membrane-bound vesicles genes (genes involved in the organization of secretory vesicles), small GTPase and peptides, plus genes associated with tight junctions and actin-mediated motility. In contrast, SCAs exhibited several features of Epithelial Mesenchymal Transition (EMT), and they preferentially expressed genes of early embryonic pituitary organogenesis and cell cycle regulators, which points to a degree of dedifferentiation of these tumors. These findings support a previous hypothesis that SCAs may originate from de-differentiated corticotrophs (6). Moreover, the researchers compared the stromal structure between SCAs and FCAs, and they found that the fibroblast subtype (adventitial fibroblasts) markers were much more abundant in the SCAs, which indicated active angiogenesis and remodeling in SCAs, considering the previously-described role of fibroblasts in modulating the tumor microenvironment and architecture (45). In FCAs, they found a higher expression of genes involved in ECM regulation, ECM receptor interaction and inflammation, and other genes for hormone discretion organization. They have noticed that in SCAs, many genes and several nuclear receptors activated by different pathways cooperate to alter stromal cell functions. scRNA-seq data analysis of this article provided insights into the main proteomic and structural differences between SCAs and FCAs, which might explain their different clinical behavior. The authors claim that the above-described notable variations in the gene signatures between SCAs and FCAs, which show a phenotypic shift in SCAs towards a mesenchymal-like cell entity, potentially give these tumors increased mobility and invasion (**Figure 3**). A scRNA-seq study published in 2024 (20) actually proposed PDZ Binding Kinase (PBK) as a new potential promoter of pituitary cells proliferation and migration, and inhibitor of pituitary cell apoptosis which indicates its role in PitNETs invasiveness (**Figure 3**). In fact, *PBK* expression was significantly associated with poor prognosis in the studied PitNETs cases.

### 4.2 Apoptosis pathways

One of the known hallmarks of all cancer types is apoptosis evasion. Cancer cells inhibit apoptotic processes, increasing the potential for mutation accumulation, angiogenesis, tumor progression and invasion, and interfering with cell differentiation (46, 47). Thus, apoptosis is considered a promising target for anticancer therapies, and research is conducted to understand intrinsic and extrinsic apoptosis pathways regulators for universal cancer therapy. With PitNETs as not an exception, many studies investigated possible regulates and pathways of apoptosis in PitNETs (48–50), although, considering the highly heterogeneous structure of these tumors, there is an actual demand for such studies at the single-cell resolution level.

A study in 2022 (51) used the scRNA-seq method to investigate apoptotic properties of pituitary tumors – Cushing’s disease. Despite the overexpression of the *PAMIP1* gene, which codes for Noxa - a protein involved in regulating cell apoptosis decisions, the researchers found an apoptotic-escape mechanism mediated by proteasome-dependent degradation of Noxa (**Figure 3**). This finding was further implanted by selected proteasome inhibition, which stabilizes Noxa function, indicating its potential role as a therapy target. In fact, this post-translational mechanism of anti-apoptotic response is also known in other tumors (52). In another study in 2024 (20), the scRNA-seq data analysis showed that both *PBK* and *GGH* genes are apoptosis-inhibitor genes (**Figure 3**).

### 4.3 Tumor recurrence

In a study in 2020 (22), researchers identified distinct markers for well and poorly-differentiated cell groups, with further validation of these markers by an IHC analysis of pituitary tissue slides in a dependent 800-patient cohort study for the same article. The data analysis showed that well-differentiated PitNETs have a noted activation of hormone biosynthesis and secretion pathways. In contrast, silent tumors, mainly with low differentiation, are characterized by upregulation of metabolism-related pathways. Interestingly, it has been shown that higher recurrence rates were observed in poorly differentiated groups of both PIT1 and TPIT lineages. In contrast, in the SF1 lineage, a higher recurrence rate was observed in well-differentiated groups. These differentiation classification findings can be applied for better recurrence prediction. This article also mentioned that the KI67 index of a tumor is affected by the tumor’s differentiation status, which could explain the conflicting results of the KI67 marker value in different PitNET studies (53–56). Thus, it is accepted that this article sets up a recurrence-evaluation system based on the molecular findings of the differentiation-related markers.

## 5 Studying the microenvironment and immune profile

It is noteworthy that not only the PitNET tumor cells should be studied but also the interactions between tumor cells themselves and between tumor cells and the tumor microenvironment (TME), which indeed affects the pathology and, thus, prognosis and outcomes of tumors. A study in 2019 (57) suggested that Tumor-Associated macrophages (TAMs) recruitment and polarization into the pro-tumoral M2 subtype drives NFPA proliferation and invasion. Another study (58) proposed a relation between lower leukocyte infiltration and the non-invasive properties of NFPAs. In addition, the EMT potential of PitNET cells is the scope of much research considering its role in tumor pathology.

Studies with the scRNA-seq method gave the potential to understand the immune profile of PitNETs on the molecular level. The study published by Zhang et al. in 2022 (26) found a heterogeneous and diverse immune microenvironment in corticotroph tumors, where immune cell distribution was relatively stable between all studied samples. The study mentioned relatively similar immune transcripts between FCAs and SCAs, although the expression of the inhibitory immunoglobulin *HAVCR2* was higher in SCAs. Researchers defined two immunosuppressive cell types, namely tumor-associated macrophages and myeloid-derived suppressor cells, where these cells might serve as therapeutic targets. A more recent study published in 2023 (23) studied PIT1-positive pituitary adenomas (PIT1-PAs) by scRNA-seq. Focusing on Tumor-associated fibroblast (TAF)-based communication networks, they clarified the essential role of IFN‐γ in TAF-mediated cellular and functional remodeling in PIT1-PA progression. It was shown that IFN‐γ-induced downregulation of Cadherin 2 (*CDH2*) reduced PAs progression, which highlighted IFN‐γ and CDH2 as potential drug targets for the aggressive PIT1-PAs. Moreover, scRNA-seq data analysis identified 3 clusters of PIT-PA stem cells. These clusters differ in their epithelial/mesenchymal hybrid status degree and immune response-ability, with a differentiation trajectory built through these stem cells clusters toward EMT-like subtypes. Additionally, this article revealed monocyte-to-TAM reprogramming in the PIT1-PA microenvironment, which was further confirmed by trajectory analysis. The filtration of natural killer T cells (NKT) was noticed by scRNA-seq results analysis of this article, and was further proven in another study (20) which showed that NK cells were likely to be highly enriched in PIT1-lineage rather than TPIT-lineage PitNETs. More active endogenous antigen-presentation on PIT1-lineage than TPIT-lineage of PitNETs, mediated by a high expression of HLA class I genes (HLA-A, HLA-B, HLA-C) in PIT1-lineage was also proposed.

Another interesting scRNA-seq study (24) aimed to investigate immune cells’ tumor diversity and composition across 23 PitNET samples categorized into the three primary lineages. This article showed three unique subtypes of tumor immune microenvironment (TIME) in PitNETs. These subtypes were identified based on the immune infiltration levels of the tumor tissue, starting from low “Immune Low” (IL) to mediate Immune Intermediate” (II) to the high infiltration subtype “Immune High” (IH). By analyzing the enriched signaling pathways in the IH group, they found that this subtype exhibited high immune activity in these tumors presented by the enriched IFN-γ response, IFN-α response, and macrophage infiltration. The data analysis showed a difference in the proportion of each immune cell subset in different lineages. For example, they found that CD4+ T cells, CD8+ T cells, and NK cells were enriched in PIT1; mast cells were increased in SF1; neutrophils were enriched in both PIT1 and SF1, while macrophages showed the highest infiltration in TPIT. To better understand these tumor immune differences, the authors of the article explored the subtypes and functions of the tumor macrophages, and they identified five macrophage subclusters distributed as follows: C1Q+ macrophages were mainly enriched in TPIT lineage tumors and mainly function in prompting protein synthesis and hormone secretion pathways; GPNMB+ macrophages were enriched in PIT1 lineage tumors and function in phosphorylation and metabolism pathways, and CX3CR1+ macrophages were enriched in SF1 lineage tumors which help in controlling tumor growth.

## 6 Limitations of scRNA-seq analysis

The power of scRNA-seq methods manifests in the level of resolution it provides for different cell and tissue types. This generates data with a high level of variability, errors, and background noise, which makes it crucial to set the optimal methodological protocols, computational tools, and annotation processes for the best data analysis and, thus, more accurate conclusions. One of the critical challenges in scRNA-seq is the need for more standardized data, especially when tested samples are highly heterogeneous, as found previously in this review for PitNET samples. Other challenges in scRNA-seq technology are low RNA input, low high-quality cell count, batch effects, protocol bias or errors in amplification and library preparation steps, lack of quality controls, and data normalization/standardization difficulties. Examples of biological challenges were previously mentioned in this review, like rare cell populations in a tissue, cell-to-cell variability, and dynamic changes in gene expression. Despite the previously mentioned obstacles in the scRNA-seq method, continuous studies using this method provide tissue-specific cell atlases, facilitating, with improved methodological protocols, the data interpretation to make scRNA-seq a more robust, trusted, and reliable tool for genomic and transcriptomic research.

## 7 Conclusion

Our review included recent peer-reviewed articles available on scientific literature databases and published between 2020 and 2024 that used the scRNA-seq method in human pituitary gland studies to provide an up-to-date revision of the data and results obtained. Nevertheless, we also read and analyzed articles from previous years when necessary to compare the scRNA-seq method findings with previous data obtained by other research methods. The articles discussed in this review, shown in **Table 1**, studied normal and pathological pituitary tissues, and shed light on some interesting differences between them. Moreover, scRNA-seq results identified novel tumor suppressor genes (*ZFP36*, *BTG1*, *DLG5*, and *ZBTB16*), cell apoptosis-inhibitor genes, genes with pro-tumoral functionality (*C1Q* in TAMs), and many cell cluster-specific genes, including tumor and stromal cell-specific marker genes. Although more scRNA-seq studies of PitNET tissues are needed to gain more knowledge and understand more details, scRNA-seq could provide some critical insights into the behavior shown in **Figure 3**. In addition to its valuable role in explaining the structures, transcriptome differences, and developmental trajectories of normal and tumor pituitary tissues, scRNA-seq helped formulate the profile of ECM and immune cells composition and interactions. However, considering the vast variability of cell types, differentiation status, and regulatory mechanisms of PitNETs, much research is needed to build a suitable reference for such studies and overcome scRNA-seq technology limitations.

**Table 1:** The table shows summarized data of scRNA-seq articles of human pituitary gland discussed in this review.

R.	Pituitary tissue Type*	PA subtype*	Sample count studied	Main Cell clusters identified
(22)	21 Human fetal pituitaries	–	21 samples	Pro.PIT1, Pre.Thyr, Pre.Som, 3 stem cell subpopulations (Stem1, Stem2, Stem3), 2 developmental status of corticotrophs (corticotrphs1, corticotroph2), Gonadotroph precursor (Pre.Gonado), 4 developmental status of gonadotrophs (gonadotroph1, gonadotroph2, gonadotroph3, gonadotroph4).
(21)	–	Somatotroph tumor (n = 4), lactotroph tumor (n = 1), thyrotroph tumor (n = 1), silent corticotroph tumors (n = 4), gonadotroph tumors (n = 8), PIT-1-positive PPA (n = 2), and PAwUIC, n = 1	23 samples from 21 patients	The normal cell types included lactotroph cells, somatotroph cells, gonadotroph cells, pituitary stem cells, endothelial cells, fibroblasts, macrophages, and T lymphocytes. The PitNETs tissues: PIT1, TPIT and SF1 tumor cell lineages, TPIT+SF1 co-expressing tumor.
(26)	–	Functioning Corticotrophs (n=5), Silent Corticotrophs (n=3), and Silent Gonadotroph (n=1) tumor samples	9 Samples	Tumor cells, stromal cells (fibroblasts and endothelial cells), immune cells, progenitor cells and a minor population of proliferating cells, 3 distinct fibroblast sub-types comprised myofibroblasts, adventitial fibroblasts and resident stromal fibroblasts. The main immune cluster comprised myeloid origin cells.
(51)	–	CD (n = 3), NFPA (n = 1), prolactinoma (n = 1), and GH-secreting adenoma (n = 1)	6 Samples	Canonical resident pituitary cell classes and circulating immune cells including erythrocytes, leukocytes, endothelial cells, pericytes, folliculostellate cells, and hormone-producing cells including corticotrophs, gonadotrophs, somatotrophs, and lactotrophs. Sub-cluster of C_Prolif_ cells within the adenoma, and a proposed folliculcytes department
(25)	3 Human adult pituitaries	Prolactinoma (n=3), (GH+PRL) plurihormonal tumor (n=2), somatotroph (n=5), thyrotroph (n=2), corticotroph (n=2), and NFPA (n=7): (1:PIT1, 2SF1, 3 TPIT and 1 null cell) tumor samples	24 Samples	In APGs: Pro.PIT1, LACTO^High^, LACTO^Medium^, PIT1_I, PIT1_II (pre lacto), Somato, Thyro, Gonado, Cortico, S100BPos Stem, S100BNeg Stem, myeloid cells, lymphocytes, fibroblasts, and endothelial cells. In PitNETs: PIT_PRL High, PIT_PRL_Medium, PIT1_ GH+PRL, PIT1_GH, PIT1_GH+CGA, PIT1_CGA, PIT1 poorly differentiated., TPIT-well differentiated, TPIT-PAX7, SF1-well/poorly differentiated., SOX2-STEM 3 stem cell subtypes in PIT-PA samples: Stem_cell_A, Stem_cell_B and Stem_cell_C, and EMT-like subtypes
(23)	–	PIT1-PAs	4 samples	Epithelial cells, immune cells, TAFs, and endothelial cells. Epithelial cells include: five differentiated cells subtypes, two EMT_like subtypes with mesenchymal signature and 3 stem cell subtypes (Stem_cell_A, Stem_cell_B and Stem_cell_C, and EMT-like subtypes)
(20)	4 Human adult pituitaries	Somatotrophs (n=4), mammosomatotrophs (n=3), lactotroph (n=3), (somato + lacto) tumor (n=1), corticotroph (n=5), null cell (n=3), thyrotroph (n=1), gonadotrophs (n=4) tumor samples	28 samples (2 samples were duplicated from 2 patients in the study)	In normal tissues: epithelial cells (stem-like cells and hormone-secreting cells), immune cells (T, B, lymphoid), stromal (fibroblast, endothelial). In tumor tissues: PIT1-lineage PitNET, SF1-lineage PitNET, TPIT-lineage PitNET, and null cell tumor, and two clusters of tumor-associated macrophages.
(27)	–	Corticotrophs (n=4)	4 samples	Corticotroph tumor cells, progenitors, stromal cells (endothelial and fibroblasts), and immune cells (T/NK cells, macrophages, and myeloid-derived suppressor cells) 3 Progenitor sub-groups were identified: epithelial progenitor, mesenchymal progenitor, and mesenchymal-epithelial transitioning cells (EMT).
(24)	–	PIT1 plurihormonal tumors (n=3), somatotroph (n=1), lactotroph (n=7) silent TPIT tumors (n=2), and 10 SF1 tumors (n=10)	23 samples	All the three lineages cells and were identified. In addition, 5 subtypes of immune cells: macrophages and DCs expressing *C1QA* and *MS4A7*, neutrophils expressing *S100A8* and *FCGR3B*, B cells expressing *MS4A1* and *CD79A*, T/NK cells expressing CD3E and NKG7, and mast cells expressing *KIT* and *MS4A2*. 2 subtypes of stromal cells: endothelial cells expressing *VWF* and *PLVAP* and fibroblasts expressing *COL1A2* and *FN1*. 3 TIME subtypes: C1QA, C1QB, and CD163- expressing macrophages.

*In this table, we included only the samples studied with scRNA-seq. Original articles might include more samples and other subtypes - for detailed information- see the cited articles.

-, no samples included; PPA, Plurihormonal pituitary adenoma; PAwUIC, Plurihormonal adenomas with unusual immunohistochemical combinations; CD, Cushing’s Disease.

## Author contributions

MU: Writing – original draft, Supervision, Writing – review & editing, Conceptualization. WA: Writing – original draft, Visualization. AS: Writing – review & editing, Conceptualization. SP: Writing – review & editing, Supervision. GM: Writing – review & editing, Project administration, Funding acquisition. NM: Writing – review & editing, Project administration, Funding acquisition.
